# Evaluation of individualized management of children with ECC after treatment under dental general anesthesia via a virtual dental home intervention: a quasi-experimental study

**DOI:** 10.3389/froh.2025.1699807

**Published:** 2025-11-05

**Authors:** Lina Dai, Tingting Wu, Xinjing Zhang, Yujia Ye, Leijiang Zhou, Yun Hu

**Affiliations:** 1 Stomatological Hospital of Chongqing Medical University, Chongqing, China; 2 Chongqing Key Laboratory of Oral Diseases and Biomedical Sciences, Chongqing, China; 3 Chongqing Municipal Key Laboratory of Oral Biomedical Engineering of Higher Education, Chongqing, China; 4 Chongqing University of Chinese Medicine, Chongqing, China; 5 Shanxi Medical University School and Hospital of Stomatology, Taiyuan, Shanxi, China

**Keywords:** individualized management, early childhood caries, dental general anesthesia, virtual dental home, caries risk assessment

## Abstract

**Introduction/objectives:**

The recurrence rate is high in children with early childhood caries (ECC) after treatment under dental general anesthesia (DGA). The purpose of this study was to evaluate the effectiveness of a virtual dental home (VDH) intervention compared to a routine intervention in the management of children with ECC following treatment under DGA, focusing on enhancing parental oral health awareness.

**Methods:**

In total, 35 child–caregiver couples presenting for treatment under DGA were randomly assigned to two groups: 19 couples in the VDH intervention group and 16 couples in the routine intervention group. The children's pre- and post-intervention number of decayed, missing, or filled teeth and caries risk assessment (CRA) were recorded during oral examinations. The participants' demographics, pre- and post-intervention Early Childhood Oral Health Impact Scale (ECOHIS) scores, the children's oral health behavior scores, and the parents' knowledge, attitudes, and practices (KAP) scores related to their children's oral health were collected through questionnaires. The length of the intervention was 2 years.

**Results:**

At baseline, no significant differences existed in demographic characteristics, CRA distribution, ECOHIS scores, the children's oral health behavior scores, or the parents' KAP scores related to their children's oral health between the two groups. At the 2-year follow-up, the ECOHIS scores significantly decreased in both groups. The mean parents' KAP and children's oral health behavior scores significantly improved in both groups, with a greater increase in the VDH intervention group. The children's CRA distribution changed significantly in the VDH intervention group compared to the routine intervention group. The caries-free rate was 47.37% in the VDH intervention group compared to 68.75% in the routine intervention group.

**Conclusions:**

The VDH intervention significantly outperformed the routine intervention method in ECC prevention and management after treatment under DGA by increasing parental oral health awareness.

**Clinical perspectives:**

This study aimed to evaluate the effectiveness of VDH intervention in preventing caries recurrence after ECC treatment under DGA. The results of this study showed that the VDH intervention, using a digital platform, was more effective in the management of ECC after treatment under DGA than the routine intervention method. These findings provide evidence for a shift toward technology-assisted models and artificial intelligence should be incorporated into the management of ECC in the future.

## Introduction

Early childhood caries (ECC) is defined as the presence of a primary tooth with one or more carious (non-cavitated or cavitated lesions), missing (due to caries), or filled surfaces in a child under the age of 6 years (1). The prevalence of ECC is 48% globally (2), and is as high as 71.9% in 5-year-old children in China (3). The complications of ECC include dental abscesses and eating difficulties, which reduce the child’s weight and body mass index for age (4). Moreover, ECC is an important predictor of dental caries in permanent dentition (5). Due to its epidemic prevalence and substantial socioeconomic burden, ECC has been identified as a priority public health problem (6), which underscores the need for advanced preventive strategies. Children's oral hygiene habits have replaced diet as the primary risk factor for ECC (7). However, we are faced with the challenge that children's oral hygiene habits are determined by their caregiver rather than children themselves to a large extent (8). Therefore, focusing on enhancing parental oral health awareness is crucial for ECC prevention and management.

Although ECC treatment based on the minimally invasive concept is accepted and carried out by many dentists, the quality of treatment involving multiple chairside appointments is often reduced due to the young age and lack of cooperation of children with ECC (9, 10). Full mouth rehabilitation under dental general anesthesia (DGA) in one single appointment is an alternative and has been reported to significantly improve children's oral health-related quality of life (OHRQoL) and promote children's catch-up growth (11, 12), achieving high parental and children's satisfaction (13). Despite the high success rate, long-term studies have shown that the recurrence rate of caries after comprehensive rehabilitation under DGA ranged from 18.8% to 79% worldwide (14–17), and recurrence has been reported to be closely related to *Streptococcus mutans* (SM) count and the caries risk assessment (CRA) score (18). In China, Tian et al. reported that among the 70 children who completed 7–13 months of follow-up in their study, 41 (59%) had caries recurrence (19). Therefore, based on the consensus of experts on ECC (20), to achieve the aim of preventing caries recurrence in children with ECC after comprehensive treatment under DGA, emphasis should be placed on enhancing parental oral health awareness and creating personalized oral health management plans based on the CRA. This approach is not only beneficial to the children's health but also reduces the economic burden on the family and society.

However, the effectiveness of routine interventions, such as group discussions and classroom teaching, may be limited due to low parental participation rates and infrequent doctor–patient interactions. The reason may be that parents play a critical role in their children's development of proper oral health-related behaviors (21). A study conducted in Beijing suggested that it is difficult to achieve this through routine oral health interventions (22); therefore, it is imperative to explore effective strategies for preventing caries recurrence among children with ECC after treatment under DGA. The concept of the “dental home” was proposed by American Academy of Pediatric Dentistry in 1993 following the medical home model (23) and does not refer to a fixed place, but rather to the ongoing relationship between the dentist and the patient, inclusive of all aspects of oral healthcare delivered in a comprehensive, continuously accessible, coordinated, and family-centered way (24). The dental home concept has proven useful for preventing ECC (25, 26), but it remains a challenge to establish a dental home for patients living in underdeveloped and developing areas with limited human resources and infrastructure. The virtual dental home (VDH) is an emerging teledentistry-based solution for CRA, prevention, and follow-up, and addresses the challenge of resource limitations in underserved areas (27–29). Despite criticisms, its potential for personalized caries management remains unexplored.

In this study, we aimed to evaluate the long-term effectiveness of individualized management of children with ECC after treatment requiring DGA by focusing on enhancing parental oral health awareness through a VDH intervention compared to a routine intervention. This may provide theoretical guidance for the prevention of caries recurrence in children with ECC after treatment under DGA.

## Materials and methods

### Sampling

A total of 38 children with ECC who visited the Department of Pediatric Dentistry from August to December 2021, and their caregivers, were recruited for this study. The inclusion criteria included pediatric patients with ECC who required full mouth rehabilitation under DGA, who strictly met the DGA indications criteria according to the Standard on Clinical Dental Treatment for Children under General Anesthesia or Sedation (30), and whose caregivers could use a cellphone. The pediatric patients were aged 2–6 based on the ECC definition and DGA indications. The exclusion criteria were pediatric patients with a history of chronic disease and mental disorder and those who had undergone prior invasive dental treatment (extractions or restorations). Informed consent was provided by the caregivers for the patient’s enrollment in the study. The 38 child and caregiver couples were randomly divided into two groups via the random number generation method using an online random number generation tool (http://sjspb.com). Thus, 19 couples were placed in the VDH intervention group and 19 in the routine intervention group. The sample size was determined by feasibility constraints (limited eligible cases meeting the strict DGA criteria), although a *post hoc* power analysis confirmed that it was adequate for detecting >30% caries recurrence reduction (*α* = 0.05, *β* = 0.2).

### Methodology

The study was conducted at the Department of Pediatric Dentistry, Stomatological Hospital of Chongqing Medical University and was approved by the Ethical Committee of Stomatological Hospital of Chongqing Medical University (CQHS-IRB-2021-34).

We used pre- and post-intervention measurements in this study. The research team consisted of two pediatric dentists, one nurse, and two pediatric dentistry graduates. Before the treatment under DGA, a link to a self-designed questionnaire was provided to the caregivers, including questions related to the participants' characteristics, Child Oral Health-Related Quality of Life (COHRQoL), the children's oral health behavior, and parental oral health awareness. COHRQoL was measured via the Early Childhood Oral Health Impact Scale (ECOHIS). Demographic information included information about the children and their main caregivers. After the 2-year intervention, the caregivers were invited to complete a questionnaire with the same content but excluding the questions related to their demographic information. The data of the questionnaire were collected by the same graduate, who was unaware of the group allocation of the participants to ensure sample blindness.

A pediatric dentist conducted the oral examinations of the pediatric patients before they underwent DGA and completed the ECC treatment under DGA based on the minimally invasive concept. A graduate recorded each patient’s decayed, missing, or filled teeth (DMFT) index and rated the pediatric patients' CRA as high, moderate, or low risk, according to the WHO criteria and the modified caries risk assessment tool (MCAT), respectively. After the 2-year intervention, the same pediatric dentist and graduate evaluated the patients’ DMFT index and CRA again. To ensure sample blindness, the dentist and graduate were unaware of the group allocation of the participants.

**Figure 1:**
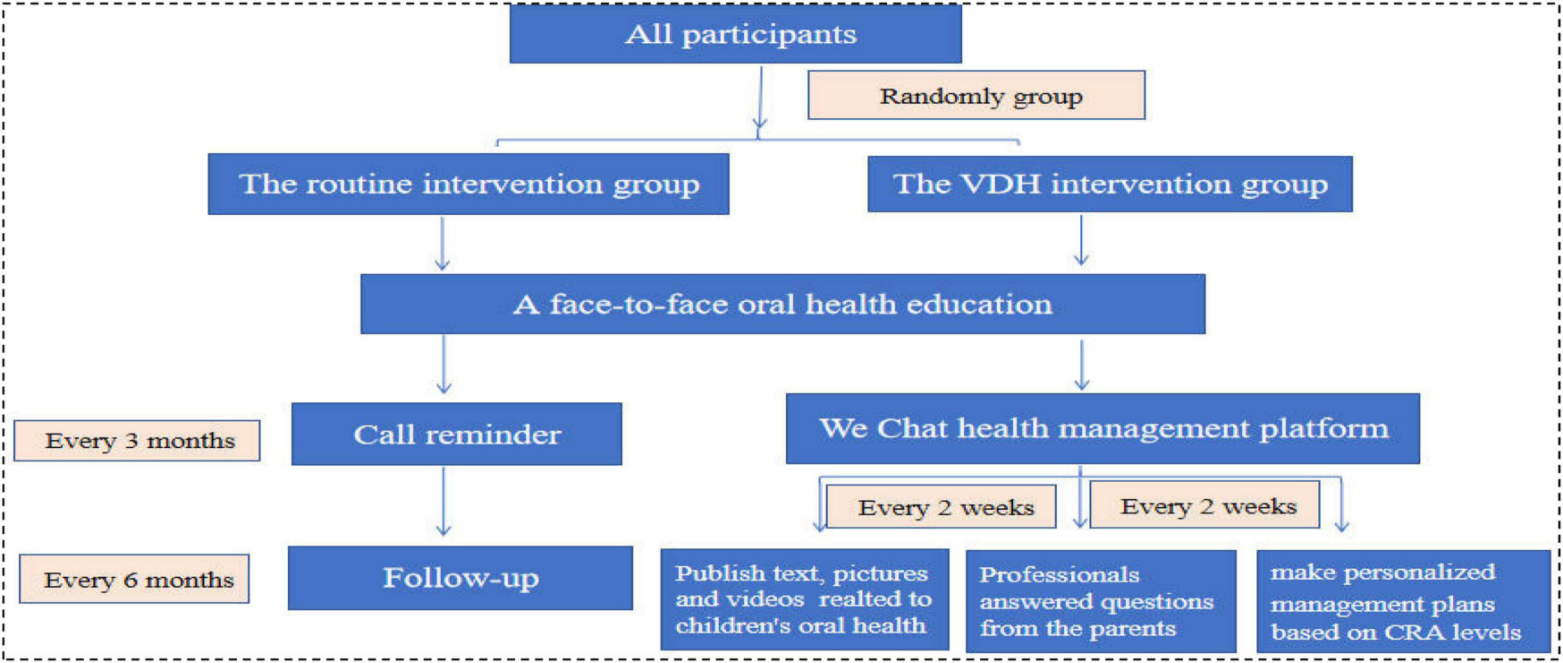
A summary of the interventions and follow-up timing of the two groups.

The intervention was implemented by another pediatric dentist and a nurse. In the routine intervention group, we ensured that each parent answered the phone successfully, and participants were excluded from the study if the parent missed more than five repeated calls or explicitly requested they be excluded. The intervention methods used in the routine intervention group were as follows (Figure 1):

1. Face-to-face oral health education immediately after the treatment under DGA, including tooth brushing, feeding, and fluoride application;
2. Telephone call reminder every 3 months that included inquiring after the children's oral health status and reminding the parents of the time of regular review;
3. Oral health education via calling the parents every 6 months, with each call lasting 5–10 min.

In the VDH group, we ensured that each parent successfully joined the WeChat management platform. The participant would be excluded from the study if the caregiver explicitly requested it. The intervention methods used in the VDH intervention group were as follows (Figure 1):

1. The same face-to-face oral health education immediately after the treatment under DGA as in the routine intervention group.
2. The caregivers (mainly parents) were invited to join a VDH health management platform based on the WeChat social software (Tencent Company, China). The nurse published educational content on children's oral health to the parents every 2 weeks and repeated this content within a week. It included prevention of ECC, brushing methods, how to choose toothpaste and toothbrushes, the sealing of 6-year molars, pit and fissure sealing, fluoride and dental floss application, space maintainers, and children's dental fears. The content was delivered in the form of text, pictures, and videos and was reviewed by the pediatric dentist.
3. Oral health Q&A: After providing the educational oral health content, the dental professionals answered questions from the parents via the platform.
4. Assistance was provided to the pediatric patients’ caregivers in creating personalized oral health management plans based on CRA levels according to the caries management protocol for 3–5-year-olds (31).

## Measurements

### Modified caries risk assessment tool (12 items)

The most commonly used caries risk assessment models include the caries risk assessment tool (CAT), the ADA caries risk model, caries management by risk assessment (CAMRA), and Cariogram (32). However, these tools are not suitable for ECC risk assessment in China due to being difficult to implement or highly sensitive (33). The MCAT for ECC used in the study, including 12 items, was designed based on CAT and factors related to ECC in China, with the advantages of having fewer items, simple content, and being easy to implement. It has been proven to predict childhood caries effectively (34).

### Chinese version of the ECOHIS (13 items)

The Chinese ECOHIS includes 13 items with minor modifications of the original version. It demonstrated acceptable validity and reliability, with a Cronbach's alpha (internal) for the total ECOHIS score of 0.91 and an intraclass correlation coefficient value (test-retest reliability) of 0.64 (35).

## Questionnaire

Studies on children's oral health behaviors and parental oral health awareness were reviewed (36–39). We initially compiled a 32-item questionnaire and invited two experts, namely, a dentist and an anesthesiologist (both with senior professional titles), to evaluate the structure and content of the questionnaire. Based on the content validity assessment conducted by the experts through face-to-face interviews, a questionnaire with 24 items was designed after deleting or modifying and adjusting the previous items. This questionnaire included 6 items on the participants' characteristics, 5 items on the children's oral health-related behaviors, and 13 items on parental oral health-related knowledge, awareness, and attitudes (KAP). One month before the formal survey, a pilot test was conducted by collecting 30 samples. Reliability and validity tests were conducted, and the Cronbach's alpha coefficient of the questionnaire was 0.749. The Kaiser–Meyer–Olkin (KMO) validity statistical test (KMO = 0.858) and Bartlett’s sphericity test (*p* < 0.0001) were conducted. An online version of the questionnaire was produced by a third-party survey company (Wenju Xing, https://www.wjx.cn) and sent to the parents in an electronic form. The formal questionnaire consisted of three parts. To avoid overlap with the clinical CRAs, the questionnaire exclusively captured parent-reported behaviors and knowledge and was structured as follows:

1. Sociodemographic characteristics (six items): Information about the children and their main caregiver(s), including the child's gender and age, parental occupations and education, and annual household income.
2. Children's oral health behavior (parent-observed, five items):The parents were invited to answer the following question: “Have you ever received oral health education for children?” (A: “Yes, I have”; B: “No, never”) at the beginning of this section. The parents were invited to write down the specific approach if they responded with A. Next, the children's oral health behavior was measured via five items on nighttime feeding, sugar consumption, frequency and duration of tooth brushing, and flossing frequency, respectively. Each item had a dichotomized outcome, with the beneficial behavior scoring 1 and the discouraged behavior scoring 0. This section’s score was calculated by summing up the scores of the items on the five behaviors (ranging from 0 to 5), with a higher score indicating a higher level of oral health behavior. These data are presented in Table 1.
3. Parents' KAP related to children's oral health (self-rated, 13 items): The first two questions were “Have you heard of the Fones tooth brushing method?” and “Have you heard of pit and fissure sealing?”. Each question had the following four possible answers: “No, I've never heard of it”, “Yes, I've heard of it, but am not familiar with it”, “Yes, I'm familiar with it, but have not applied it to my child”, and “Yes, and I've applied it to my child”, which were scored 0, 1, 2, and 3, respectively. The other 11 questions had 2 possible answers, namely, “agree” or “disagree”, which were scored 1 when answered correctly or 0. This section’s score ranged from 0 to 17, with a higher score indicating the parent had a higher level of knowledge, awareness, and attitudes related to children's oral health. These data are presented in Table 2.

**Table 1:** Results of the changes in the children's oral health behavior during the 2 years of follow-up.

Item	Answer (score)	VDH intervention group		Routine intervention group		Before the intervention		After the intervention	
		Before intervention	After intervention	Before intervention	After intervention	VDH intervention group	Routine intervention group	VDH intervention group	Routine intervention group
		(*n* = 19)	(*n* = 19)	(*n* = 16)	(*n* = 16)	(*n* = 19)	(*n* = 16)	(*n* = 19)	(*n* = 16)
Nighttime feeding	Yes (0)	11 (57.89%)	4 (21.05%)	13 (81.25%)	4 (25.00%)	11 (57.89%)	13 (81.25%)	4 (21.05%)	4 (25.00%)
	No (1)	8 (42.11%)	15 (78.95%)	3 (18.75%)	12 (75.00%)	8 (42.11%)	3 (18.75%)	15 (78.95%)	12 (75.00%)
	*χ*²	5.397		10.165		2.198		0.077	
	*p*	0.020*		0.001**		0.138		0.782	
Sugar intake	Daily or several times a week (0)	5 (26.32%)	5 (26.32%)	7 (43.75%)	8 (50.00%)	5 (26.32%)	7 (43.75%)	5 (26.32%)	8 (50.00%)
	Rarely (1)	14 (73.68%)	14 (73.68%)	9 (56.25%)	8 (50.00%)	14 (73.68%)	9 (56.25%)	14 (73.68%)	8 (50.00%)
	χ²	0		0.125		1.172		2.087	
	*p*	1		0.723		0.279		0.149	
Frequency of tooth brushing	≦once a day (0)	5 (26.32%)	0 (0.00%)	8 (50.00%)	10 (62.50%)	5 (26.32%)	8 (50.00%)	0 (0.00%)	10 (62.50%)
	≥twice a day (1)	14 (73.68%)	19 (100.00%)	8 (50.00%)	6 (37.50%)	14 (73.68%)	8 (50.00%)	19 (100.00%)	6 (37.50%)
	χ²	5.758		0.508		2.087		16.625	
	*p*	0.016*		0.476		0.149		0.000**	
Duration of tooth brushing	≦2 min (0)	14 (73.68%)	12 (63.16%)	13 (81.25%)	9 (56.25%)	14 (73.68%)	13 (81.25%)	12 (63.16%)	9 (56.25%)
	≥3 min (1)	5 (26.32%)	7 (36.84%)	3 (18.75%)	7 (43.75%)	5 (26.32%)	3 (18.75%)	7 (36.84%)	7 (43.75%)
	χ²	0.487		2.327		0.282		0.173	
	*p*	0.485		0.127		0.595		0.678	
Flossing frequency	Never (0)	18 (94.74%)	17 (89.47%)	15 (93.75%)	15 (93.75%)	18 (94.74%)	15 (93.75%)	17 (89.47%)	15 (93.75%)
	Once or more a week (1)	1 (5.26%)	2 (10.53%)	1 (6.25%)	1 (6.25%)	1 (5.26%)	1 (6.25%)	2 (10.53%)	1 (6.25%)
	χ²	0.362		0		0.016		0.203	
	*p*	0.547		1		0.9		0.653	

* Indicated *P*<0.05.

** Indicated *P*<0.01.

**Table 2:** Results of the changes in the parents' knowledge, awareness, and attitudes related to their children's oral health during the 2-year follow-up.

Item	Score	VDH intervention group		Routine intervention group		Before intervention		After intervention	
		Before intervention	After intervention	Before intervention	After intervention	VDH intervention group	Routine intervention group	VDH intervention group	Routine intervention group
Have you heard of the Fones brushing method?	0	12 (63.16%)	3 (15.79%)	11 (68.75%)	6 (37.50%)	12 (63.16%)	11 (68.75%)	3 (15.79%)	6 (37.50%)
	1	4 (21.05%)	5 (26.32%)	4 (25.00%)	5 (31.25%)	4 (21.05%)	4 (25.00%)	5 (26.32%)	5 (31.25%)
	2	1 (5.26%)	5 (26.32%)	1 (6.25%)	4 (25.00%)	1 (5.26%)	1 (6.25%)	5 (26.32%)	4 (25.00%)
	3	2 (10.53%)	6 (31.58%)	0 (0.00%)	1 (6.25%)	2 (10.53%)	0 (0.00%)	6 (31.58%)	1 (6.25%)
	χ²	10.178		4.382		1.8		4.458	
	*p*	0.017*		0.223		0.615		0.216	
Have you heard of pit and fissure sealing?	0	7 (36.84%)	0 (0.00%)	5 (31.25%)	0 (0.00%)	7 (36.84%)	5 (31.25%)	0 (0.00%)	0 (0.00%)
	1	7 (36.84%)	0 (0.00%)	5 (31.25%)	0 (0.00%)	7 (36.84%)	5 (31.25%)	0 (0.00%)	0 (0.00%)
	2	5 (26.32%)	5 (26.32%)	5 (31.25%)	6 (37.50%)	5 (26.32%)	5 (31.2%5)	5 (26.32%)	6 (37.50%)
	3	0 (0.00%)	14 (73.68%)	1 (6.25%)	10 (62.50%)	0 (0.00%)	1 (6.25%)	14 (73.68%)	10 (62.50%)
	χ²	28		17.455		1.42		0.504	
	*p*	0.000**		0.001**		0.701		0.478	
Bacteria are one of the main factors leading to dental caries	0	0 (0.00%)	1 (5.26%)	2 (12.50%)	0 (0.00%)	0 (0.00%)	2 (12.50%)	1 (5.26%)	0 (0.00%)
	1	19 (100.00%)	18 (94.74%)	14 (87.50%)	16 (100.00%)	19 (100.00%)	14 (87.50%)	18 (94.74%)	16 (100.00%)
	χ²	1.027		2.133		2.519		0.867	
	*p*	0.311		0.144		0.112		0.352	
Children's gums bleeding is normal when brushing teeth	0	1 (5.26%)	0 (0.00%)	4 (25.00%)	1 (6.25%)	1 (5.26%)	4 (25.00%)	0 (0.00%)	1 (6.25%)
	1	18 (94.74%)	19 (100.00%)	12 (75.00%)	15 (93.75%)	18 (94.74%)	12 (75.00%)	19 (100.00%)	15 (93.75%)
	χ²	1.027		2.133		2.763		1.222	
	*p*	0.311		0.144		0.096		0.269	
Fluoride is an effective way to prevent caries	0	3 (15.79%)	4 (21.05%)	3 (18.75%)	3 (18.75%)	3 (15.79%)	3 (18.75%)	4 (21.05%)	3 (18.75%)
	1	16 (84.21%)	15 (78.95%)	13 (81.25%)	13 (81.25%)	16 (84.21%)	13 (81.25%)	15 (78.95%)	13 (81.25%)
	χ²	0.175		0		0.054		0.029	
	*p*	0.676		1		0.817		0.865	
Fissure sealing should be considered to prevent caries.	0	3 (15.79%)	1 (5.26%)	1 (6.25%)	3 (18.75%)	3 (15.79%)	1 (6.25%)	1 (5.26%)	3 (18.75%)
	1	16 (84.21%)	18 (94.74%)	15 (93.75%)	13 (81.25%)	16 (84.21%)	15 (93.75%)	18 (94.74%)	13 (81.25%)
	χ²	1.118		1.143		0.781		1.561	
	*p*	0.29		0.285		0.377		0.212	
Toothbrush, toothpaste, and brushing method can all affect the effectiveness of brushing	0	1 (5.26%)	1 (5.26%)	2 (12.50%)	1 (6.25%)	1 (5.26%)	2 (12.50%)	1 (5.26%)	1 (6.25%)
	1	18 (94.74%)	18 (94.74%)	14 (87.50%)	15 (93.75%)	18 (94.74%)	14 (87.50%)	18 (94.74%)	15 (93.75%)
	χ²	0		0.368		0.58		0.016	
	p	1		0.544		0.446		0.9	
Dental floss should be used to prevent ECC	0	8 (42.11%)	5 (26.32%)	6 (37.50%)	6 (37.50%)	8 (42.11%)	6 (37.50%)	5 (26.32%)	6 (37.50%)
	1	11 (57.89%)	14 (73.68%)	10 (62.50%)	10 (62.50%)	11 (57.89%)	10 (62.50%)	14 (73.68%)	10 (62.50%)
	χ²	1.052		0		0.077		0.504	
	*p*	0.305		1		0.782		0.478	
Children should have regular oral health examinations	0	3 (15.79)	2 (10.53)	0 (0.00)	1 (6.25)	1 (5.26)	0 (0.00)	0 (0.00)	1 (6.25)
	1	16 (84.21%)	17 (89.47%)	16 (100.00%)	15 (93.75%)	18 (94.74%)	16 (100.00%)	19 (100.00%)	15 (93.75%)
	χ²	0.23		1.032		0.867		1.222	
	*p*	0.631		0.31		0.352		0.269	
The “6-year molar” is the first permanent molar; once it erupts, it needs to be sealed promptly	0	5 (26.32%)	0 (0.00%)	5 (31.25%)	3 (18.75%)	5 (26.32%)	5 (31.25%)	0 (0.00%)	3 (18.75%)
	1	14 (73.68%)	19 (100.00%)	11 (68.75%)	13 (81.25%)	14 (73.68%)	11 (68.75%)	19 (100.00%)	13 (81.25%)
	χ²	5.758		0.667		0.104		3.896	
	*p*	0.016*		0.414		0.748		0.048*	
Primary teeth will be replaced by permanent teeth; no repair is necessary until the child feels pain	0	3 (15.79%)	2 (10.53%)	0 (0.00%)	3 (18.75%)	3 (15.79%)	0 (0.00%)	2 (10.53%)	3 (18.75%)
	1	16 (84.21%)	17 (89.47%)	16 (100.00%)	13 (81.25%)	16 (84.21%)	16 (100.00%)	17 (89.47%)	13 (81.25%)
	χ²	0.23		3.31		2.763		0.48	
	*p*	0.631		0.069		0.096		0.489	
It is necessary to treat caries in primary teeth or they may damage the successor permanent teeth	0	2 (10.53%)	0 (0.00%)	2 (12.50%)	3 (18.75%)	0 (0.00%)	2 (12.50%)	2 (10.53%)	3 (18.75%)
	1	17 (89.47%)	19 (100.00%)	14 (87.50%)	13 (81.25%)	19 (100.00%)	14 (87.50%)	17 (89.47%)	13 (81.25%)
	χ²	2.111		0.237		2.519		0.48	
	*p*	0.146		0.626		0.112		0.489	
Good oral habits are important for children's health, but these oral habits are cultivated by relying on themselves rather than their parents	0	11 (57.89%)	15 (78.95%)	14 (87.50%)	16 (100.00%)	11 (57.89%)	14 (87.50%)	15 (78.95%)	16 (100.00%)
	1	8 (42.11%)	4 (21.05%)	2 (12.50%)	0 (0.00%)	8 (42.11%)	2 (12.50%)	4 (21.05%)	0 (0.00%)
	χ²	1.949		2.133		3.73		3.803	
	*p*	0.163		0.144		0.053		0.051	

* Indicated *P*<0.05.

** Indicated *P*<0.01.

### Statistical analysis

The statistical analyses were performed using SPSS version 28.0 (IBM Corp., Armonk, NY, USA). Continuous variables with normal distribution are expressed as mean ± standard deviation (SD), while non-normally distributed data are reported as median (interquartile range, IQR). Categorical variables are summarized as frequencies and percentages. The following group comparisons were employed: independent Student's *t*-tests for normally distributed continuous variables between two groups, chi-square tests (or Fisher's exact tests for expected cell counts <5) for categorical variables, and Mann–Whitney *U* tests for non-parametric continuous data. Statistical significance was defined as two-sided *P* < 0.05.

## Results

At baseline, 38 caregiver–child couples (VDH group: *n* = 19; routine group: *n* = 19) were enrolled. After 2 years of follow-up, 3 couples in the routine intervention group explicitly requested to withdraw from the study due to their own reasons, leaving a total of 35 couples (VDH group: *n* = 19; routine group: *n* = 16) remaining. Thus, the response rate was 92.11%. Two-thirds of the children were boys, and the mean age of the children in the VDH and routine intervention groups was 4.67 ± 1.74 and 4.01 ± 0.90, respectively. The characteristics of the 35 caregiver–child couples are presented in Table 3. There was no statistically significant difference in the demographic characteristics between the two groups (*p* > 0.05).

**Table 3:** Characteristics of study participants.

Variables	Subgroups	VDH intervention group	Routine intervention group	*Χ*^2^/t	*p*
		*n* = 19	*n* = 16		
Age		4.67 ± 1.74	4.01 ± 0.90	1.442	0.161
Gender	Boy	12 (63.16%)	9 (56.25%)	0.173	0.678
	Girl	7 (36.84%)	7 (43.75%)		
Occupation of the father	Managerial or professional	5 (26.32%)	3 (18.75%)	1.446	0.695
	Labor	8 (42.11%)	5 (31.25%)		
	Clerical	3 (15.79%)	5 (31.25%)		
	Freelance work	3 (15.79%)	3 (18.75%)		
Occupation of the mother	Managerial or professional	3 (15.79%)	3 (18.75%)	1.868	0.6
	Labor	10 (52.63%)	6 (37.50%)		
	Clerical	1 (5.26%)	3 (18.75%)		
	Freelance work	5 (26.32%)	4 (25.00%)		
Education of the father	Above bachelor’s degree	12 (63.16%)	8 (50.00%)	0.614	0.433
	Below bachelor’s degree	7 (36.84%)	8 (50.00%)		
Education of the mother	Above bachelor’s degree	12 (63.16%)	7 (43.75%)	1.318	0.251
	Below bachelor’s degree	7 (36.84%)	9 (56.25%)		
Annual household income	≥￥5,000	16 (84.21%)	14 (87.50%)	0.077	0.782
	<￥5,000	3 (15.79%)	2 (12.50%)		

**Figure 2:**
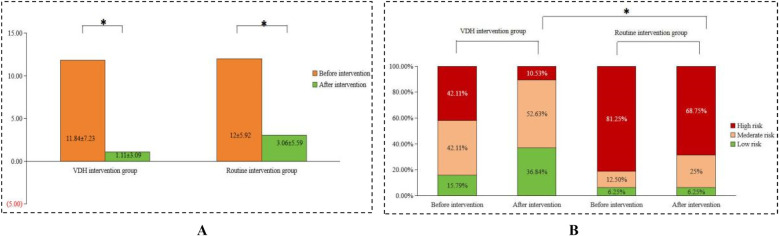
Comparison of **(A)** the ECOHIS score and **(B)** CRA level before and after the interventions between the VDH intervention and routine intervention groups.

Before the interventions, there was no significant difference (*p* > 0.05) in the ECOHIS scores between the two groups of children. After the interventions, the ECOHIS scores decreased significantly in both groups (*p* < 0.05), with a greater decrease in the VDH group (Δ = −8.2 vs. −5.1, *p* < 0.05). Compared to the routine intervention group, the VDH group had a more significant decrease, as shown in Figure 2A. The CRA levels in the VDH and routine intervention groups were not significantly different pre-intervention. However, the difference in CRA level between the two groups was significant post-intervention (*p* < 0.05), as shown in Figure 2B.

**Figure 3:**
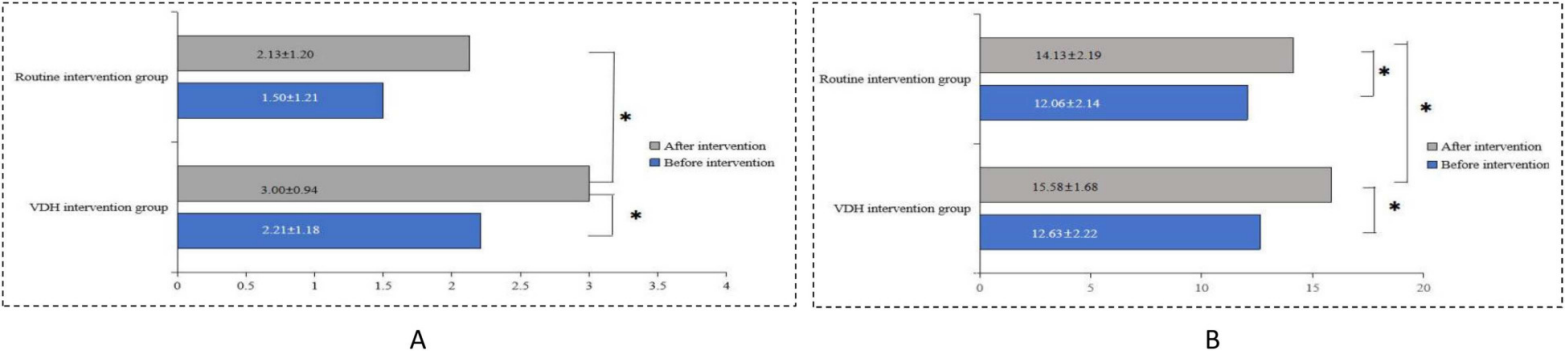
The mean **(A)** children's oral health behavior and **(B)** parents' knowledge, awareness, and attitudes scores before and after the interventions.

At baseline, there was no significant difference in the mean children's oral health behavior scores between the two groups (*p* > 0.05). At the 2-year follow-up, the mean score in both groups increased; however, the mean score in the VDH intervention group was significantly higher than that of the routine intervention group (3.00 ± 0.94 vs. 2.13 ± 1.20, *p* < 0.05). In addition, the mean score in the VDH intervention group changed significantly after the intervention (3.00 ± 0.94 vs. 2.21 ± 1.18, *p* < 0.05), as shown in Figure 3A. Specifically, compared to the routine intervention group, the frequency of tooth brushing changed significantly after the intervention in the VDH intervention group (*p* < 0.05). Furthermore, compared to before the intervention, nighttime feeding changed significantly in the VDH intervention group after the intervention (*p* < 0.05), as shown in Table 3.

Before the intervention, there was no significant difference between the two groups in the mean parents' knowledge, awareness, and attitude scores related to children's oral health (*p* > 0.05).

After the intervention, the mean score in both groups increased significantly (*p* < 0.05), and the improvement in the VDH intervention group (from 12.63 ± 2.22 to 15.84 ± 1.68) was more significant than that in the routine intervention group (from 12.06 ± 2.14 to 14.13 ± 2.19) (Figure 3B). Specifically, the parents' recognition of “pit and fissure sealing” significantly changed in the routine intervention group at the 2-year follow-up; furthermore, besides “pit and fissure sealing,” parents' recognition of “tooth brushing method,” and “the six-year molar” also significantly changed in the VDH intervention group (Table 2).

## Discussion

This study evaluated the effectiveness of a 2-year VDH intervention compared to routine methods in managing ECC following comprehensive treatment under DGA. Our findings demonstrate that the VDH approach, which emphasizes increasing parental oral health awareness through digital platforms, yielded superior outcomes in both the children's oral health behaviors and parents' knowledge and attitudes compared to the conventional interventions.

The VDH intervention group showed particularly promising results, with 47.37% of the children remaining caries-free during the 2-year observation period. This surpasses the 41% caries-free rate reported in a previous Chinese study with only 13 months of follow-up (19), suggesting that the VDH model may provide more sustainable prevention benefits. The success of this digital approach aligns with current trends in pediatric dentistry that emphasize technology-assisted, family-centered care models (40).

Our results revealed significant enhancements in several critical areas of parental oral health literacy through the VDH intervention. The VDH group showed marked improvement in adoption of the Fones brushing method (from 63.16% to 84.21% awareness), while the routine methods showed minimal change. This demonstrates the effectiveness of digital platforms in teaching proper brushing techniques through visual demonstrations and regular reinforcement. In addition, both interventions improved the caregivers’ knowledge about pit and fissure sealing, but those in the VDH group achieved universal recognition (100%) of the importance of sealing 6-year molars promptly. This universal recognition in the VDH group highlights the advantage of persistent digital reminders and educational content delivery. Furthermore, while both groups reduced nighttime feeding [a known caries risk factor (23)], only the VDH group maintained significant improvements in brushing frequency. This sustained behavioral modification underscores the value of continuous digital engagement compared to sporadic in-person education.

Despite these successes, our study identified areas requiring additional focus. First, neither intervention significantly improved flossing practices, with only 5.26% of the parents in the VDH group and 6.25% in the routine intervention group reporting regular flossing. However, the use of dental floss should never be discouraged, as it is effective in reducing proximal caries in primary dentition (41). This finding highlights the need for more innovative approaches to flossing education, potentially incorporating augmented reality demonstrations or gamified learning modules.

In addition, sugar consumption patterns remained largely unchanged in both groups, as at least half or more of the children in each group consumed sugar daily or several times a week. These findings, while in contrast to the American Academy of Pediatric Dentistry's recommendation to prevent early childhood caries, which advises against the frequent consumption of sugar-sweetened liquids and/or solid foods (42), were consistent with a report by Razeghi et al. (43). This reflects the complex interplay between family dietary habits and children's oral health, indicating that future interventions may need to incorporate nutritional counseling for the whole family.

The significant reduction in the ECOHIS scores in both groups (*p* < 0.05) confirms previous findings that comprehensive treatment under DGA, coupled with postoperative interventions, can yield lasting quality of life improvements (44–46). However, the VDH group showed more substantial and sustained improvements, suggesting that continuous digital support may enhance and prolong these benefits.

The VDH intervention demonstrated remarkable success in caries risk reduction, with the proportion of high-risk children decreasing from 31.58% to 10.53% and 63.16% of the participants showing an improvement in at least one risk category. These results compare favorably to recent studies using similar digital interventions (47, 48) and support the growing evidence for technology-assisted caries management in pediatric populations.

The success of the VDH model suggests several important implications for clinical practice. First, digital platforms can effectively deliver sustained oral health education. Second, continuous remote monitoring improves compliance with preventive measures. Third, personalized content delivery enhances knowledge retention. Future interventions should incorporate family-centered nutritional guidance, innovative flossing education tools, artificial intelligence-assisted risk assessment, and automated reminders and progress tracking into their design.

While this study had promising results, several limitations should be acknowledged.

First, the relatively small sample size may limit the generalizability of our findings. Future studies with larger cohorts would strengthen the evidence base. Second, the 2-year follow-up period, while providing valuable intermediate-term data, may be insufficient to assess long-term caries prevention outcomes. Longitudinal studies spanning 5+ years would be ideal. Third, the VDH platform relied exclusively on WeChat, which may not be accessible to all populations. Future iterations should incorporate multi-platform accessibility. Fourth, the outcome measures relied heavily on self-reported behaviors, and potential confounding variables, such as socioeconomic status or parental education level, which may influence oral health behaviors and are subject to recall bias, were not taken into account. Future studies could incorporate objective measures such as plaque indices. In addition, the study was conducted at a single academic center, potentially limiting the external validity. A multicenter design would enhance applicability across different clinical settings.

## Conclusion

Our 2-year follow-up study found that the VDH intervention model significantly outperformed routine methods in maintaining oral health following comprehensive ECC treatment under DGA. By leveraging digital technologies to enhance parental education and engagement, this approach achieved superior outcomes in caries prevention, risk reduction, oral health behaviors, and quality of life improvements. These findings support the growing shift toward technology-assisted, family-centered care models in pediatric dentistry. Future research should focus on optimizing digital platforms to address persistent challenges in flossing adherence and dietary modifications.

## Data Availability

The original contributions presented in the study are included in the article/Supplementary Material, further inquiries can be directed to the corresponding author.
